# Supplementary material for: Are carbon emissions trading and green financial instruments synergistic? -Comprehensive quantitative research based on content analysis
Source: PLoS One. 2024 Mar 7;19(3):e0298601. doi: 10.1371/journal.pone.0298601 (PMC10919601; doi:10.1371/journal.pone.0298601)
Supplement: S1 Data — (ZIP) [file pone.0298601.s001.zip › Data/地级市Z_F工作报告.zip!/╨┬╜«/┐╦└¡┬Ω╥└╩╨/└┤╫╘╣1⁄2╓┌║┼í╛┬φ┐╦╩2╛▌═°í┐_2016.docx]

各位代表：

现在，我代表市人民政府向大会作政府工作报告，请予审议，并请市政协委员和其他列席人员提出意见。

过去五年和2015年的工作回顾

过去的五年，是克拉玛依打造世界石油城迈出坚实步伐的五年。五年来，我们坚决贯彻中央、自治区关于新疆工作的重大决策，全面落实市委各项部署，坚定打造世界石油城战略目标不动摇，妥善应对复杂形势和风险挑战，凝心聚力，抢抓机遇，聚焦城市转型和可持续发展，突出创新引领，各项事业取得了积极成效，“十二五”规划确定的目标基本实现。

——过去的五年，全市经济保持平稳运行，发展质量明显提升。初步测算，全市地区生产总值年均增长3.6%，一般公共预算收入年均增长12.2%，社会消费品零售总额年均增长11.1%。地方经济发展质量显著提高，扣除中央石油石化业贡献份额，全市地方生产总值由“十一五”末的95亿元，增长到176.5亿元，占GDP的比重由13.4%提高到26.3%。产业结构进一步优化，信息、金融、旅游三大新兴产业年均增速达到19%，第三产业占GDP的比重由“十一五”末的9.8%提高到19.8%（老口径），显示出强劲发展势头。

——过去的五年，城市现代化品质逐步优化，城市功能日趋完善。围绕打造高品质城市，完成了克—白组团整体规划，加大基础设施和生态环境建设力度，克拉玛依河改造及文体中心、会展中心、大学城、克一号井等一批基础性、标志性、功能性项目建成投用，城市功能不断完善。城市精细化管理“大联动”全面启动，持续推进“大绿化工程”和“蓝天工程”，全市绿地面积由“十一五”末的3.3万亩提高到10.2万亩，人均公园绿地面积由“十一五”末的9平方米提高到11.5平方米，城市环境更加秀美宜居。

——过去的五年，市民生活水平不断提高，各项社会事业蓬勃发展。坚持民生优先不动摇、民生投入不削减，5年用于民生的支出累计达到300亿元，占公共财政支出的近80%。城镇居民人均可支配收入和农牧民人均纯收入年均增长14%和13%，分别达到33430元和19000元；城镇登记失业率始终控制在3%以内。坚持不懈办好教育、医疗、就业等民生大事，大力发展各项社会事业，持续强化食品药品安全监管，着力解决与百姓生活密切相关的问题，市民的自豪感、幸福感、归属感不断增强。蝉联“全国文明城市”称号。

——过去的五年，安全基础日益牢固，社会大局保持和谐稳定。

——过去的五年，对外交流合作不断加强，城市影响力不断扩大。与亚欧、北美等国家的15个城市建立了友好关系。成功举办了中巴经济走廊论坛、中国（克拉玛依）国际石油天然气及石化技术装备展览会、信息化创新克拉玛依国际学术论坛等国际性会展活动，加入了世界能源城市伙伴组织，城市对外合作水平和国际知名度逐步提升。深化兵地油融合发展，以“四地五师”为主体的北疆西部区域融合发展格局初步形成。

过去的五年，是我市经济社会发展最好、城市转型推进最快、生态环境变化最大、群众得实惠最多的时期。这些成绩的取得，是自治区党委、政府、中石油集团公司关心支持和市委正确领导的结果，是全市人民开拓进取、真抓实干的结果，是市人大及其常委会有效监督、市政协积极参政议政的结果，是驻市中央企业与地方发挥体制优势，步调一致谋划发展、推动发展的结果，是所有关心支持克拉玛依发展的驻市各单位、兵团团场、社会团体、友好人士、离退休老同志，以及在克拉玛依投资置业、务工经商人士共同努力的结果，是驻市人民解放军、武警官兵，全市公安干警、广大民兵无私奉献的结果。在此，我代表市人民政府表示最诚挚的感谢和最崇高的敬意！

五年的成绩来之不易，积累的经验弥足珍贵。我们最深刻的体会是：必须坚持在市委的坚强领导下，树立先进的发展理念，以开放的视野和包容的胸怀，面向世界学习、面向未来发展，形成具有克拉玛依特色的可持续发展之路；必须坚持一张蓝图绘到底，接力奋进、一以贯之抓好落实；必须坚持改革创新驱动，着力破解发展中的各种问题和困难；必须坚持以人为本，让全市人民共享发展改革成果；必须坚持社会稳定和长治久安，以安全环境保障发展；必须坚持“两个可持续”，做到经济发展与环境保护并重；必须发挥特殊体制优势，树立全市一盘棋的思想，共同建设美好家园。

刚刚过去的2015年，面对经济下行压力增大、国际原油价格下跌、炼化企业停产检修、企业利润下滑和市场预期不旺等因素影响，我们坚定打造世界石油城战略不动摇，以深化改革和科技创新为动力，坚持稳增长、调结构，制定并实施了应对经济下行的7个方面27条具体措施，确定了稳增长、促发展的117条工作要点，狠抓推进落实，较好地实现了全年预期目标，全市呈现出经济发展提质、城市品质提升、民生有效保障、社会安全稳定的良好局面。

初步统计，2015年全市实现地区生产总值670.1亿元，增长0.5%（按可比价计算，下同），扣除中央石油石化业贡献份额，实现地方生产总值176.5亿元，增长7.3%。实现一般公共预算收入75亿元，增长2%；支出89.7亿元，下降4.5%。完成固定资产投资305.6亿元，下降8.7%。实现社会消费品零售总额58.8亿元，增长5.4%。居民消费价格指数上涨0.4%。城镇居民人均可支配收入33430元，增长10.5%；农牧民人均纯收入19000元，增长9.2%。城镇登记失业率控制在1%以内。

（一）六大基地建设平稳有序。全年生产原油1282万吨，天然气31.4亿立方米，辖区内原油产量988万吨。其中，新疆油田公司生产原油1180万吨，辖区内产量896万吨；新春采油厂生产原油102万吨，辖区内产量92万吨。油砂矿采矿权获得自治区批准，实现了地方在非常规能源开发上的突破。完成了克石化公司属地法人注册与合资合作改革，两大石化公司全年加工原油1221.6万吨。成立了新疆石油工程技术服务企业联合会，新疆石油工程技术服务企业资质认定管理中心获自治区批准设立，与人民银行乌鲁木齐中心支行签订了金融支持新疆石油工程技术服务产业战略合作协议，搭建了服务企业发展的新平台。贝肯、正通等地方技术服务企业已分别在哈萨克斯坦、蒙古、吉尔吉斯斯坦等国成功参与油田开发项目，正在由单一技术服务型向综合石油公司转型。新油国际油管再制造、永升聚元装备制造产业园等一批项目投产或开工建设，机械装备制造产业链进一步完善。教育部正式批准中国石油大学（北京）在克拉玛依建设校区，成为新疆首所教育部直属高等院校。厚博学院迁建工作进展顺利，首批学生已于去年9月入学。

（二）三大新兴产业发展势头良好。华为云服务数据中心、中石油数据中心（克拉玛依）投入使用，国家“一带一路”大数据中心、“天地图·中亚空间信息枢纽”项目落户我市。昆仑银行设立了北京国际业务结算中心，伊犁分行已获批筹，中石油专属财产保险公司海外业务进一步拓展。中亚商品交易中心成品油直批业务上线交易，进一步拓宽了交易渠道。资本市场发展迅速，12家地方企业成功登陆全国股转系统。魔鬼城创建国家5A级旅游景区积极推进，克一号井、百里油区等石油工业旅游项目基本建成。自驾游游客占到全市接待游客总量的57.8%，成为旅游产业发展的新亮点。2015年，信息、金融、旅游三大新兴产业增速均达到13%以上。

（三）农业现代化建设稳步推进。国家级现代农业示范区和国家农业科技园区分别获得农业部、科技部认定。现代畜牧业产业园建设全面启动，绿成乳品厂迁建投产，一批标准化肉羊肉牛养殖场和养殖专业合作社加快建设。农业综合开发区和白碱滩区全部退出棉花种植，5万亩优质饲草料基地初步建成，蔬菜种植标准化、现代化水平不断提升，鼠标购物和订单配送等农产品营销新业态加快发展。

（四）改革与创新工作扎实有效。坚持问题导向，围绕央企属地法人注册与合资合作、政府与社会资本合作、工商注册登记便利化等工作，大力推进经济领域综合改革。以转变职能、简政放权、加强监管为核心，加快了行政管理体制改革。教育、就业、医疗、养老等各项社会事业改革扎实推进。文化、生态文明、新型城镇化建设等领域的改革步伐进一步加快。坚持创新驱动，与中科院大连化物所合作，共建先进能源化工及清洁生产技术创新中心、大连化物所国家技术转移中心克拉玛依中心和清洁能源国家实验室中试基地，为企业创新发展搭建了服务平台，增强了城市创新发展能力。充分发挥政策资金引导作用，积极开展“拨改投”试点，有154家企业、212个项目获得国家、自治区和我市近1亿元专项资金支持，企业科技创新能力不断增强。

（五）对外开放与合作交流继续加强。高质量举办了中巴经济走廊论坛，在基础设施建设和民生等领域签署合作协议20项，涉及金额103.5亿元。深化友城合作，先后与巴基斯坦瓜达尔区、蒙古乌兰巴托市缔结为友好城市，城市对外合作水平和国际知名度逐步提升。首批境外联络处在巴基斯坦伊斯兰堡、卡拉奇、瓜达尔成立，为企业开展经贸合作提供了便利。举办了第五届北疆西部区域融合发展研讨会，各方共达成23项合作协议，区域互惠互利和共同发展的格局逐步形成。

（六）城市现代化品质进一步提升。《克拉玛依市城市总体规划（2014—2030年）》获自治区批准，开展了16项控制性详细规划编制，城市规划体系更加完善。完成了金龙湖、独山子展览（博物）馆、白碱滩体育健身中心、乌尔禾10KV供电线路改造工程等重点项目建设，城市功能进一步完善。城市管理“大联动”工作全面启动，初步建立了精细化管理标准考核体系。以自治区成立60周年和克拉玛依油田发现60周年为契机，开展了市容市貌整治、白色垃圾清理、餐饮业油烟污染治理等环境整治专项行动。持续推进生态环境建设，开展了 “大绿化工程”和“蓝天工程”建设，全年新增绿地7811亩，新增苗圃500亩，全市空气质量优良率达91.5%，较上年提高4.1个百分点。

（七）各项事业和民生工作持续强化。进一步强化区级教育管理职能，完成了市属五中、十中、十三中移交白碱滩区管理。从去年秋季开始，小学起始年级全部实行了民汉学生统一编班。2015年全市高考民汉综合上线率99.3%，增长1.3个百分点。确定公立医院改革实施方案，各项工作有序推进。建立了96565预约诊疗系统，进一步方便了群众就医。重大疾病预防控制工作继续加强，食品安全溯源体系初具成效。出台了新形势下促进就业和大众创业、万众创新的一系列政策措施，进一步激发了全社会创新创业活力，促进了就业。国家公共文化服务体系示范区创建工作进展顺利，文化馆、科技馆相继建成投用。举办了新疆国际民族舞蹈节等一系列大型文体活动，群众多层次、多元化的文体需求得到满足。全年新开工保障房3448套，完成“大西沟”、“西大沟”片区的棚户区拆迁与安置，市民居住环境进一步改善。进一步强化安全生产监督管理，开展了以道路交通、危险化学品、城镇燃气等行业（领域）为重点的安全生产专项整治，确保了全市安全生产形势持续稳定。

（八）社会大局保持安全稳定。严格治安重点要素管控和要害部位防范，全市刑事案件发案率降低了3.8%，破案率提高了1.6%。在全国率先建立了个人诉讼保险制度，引导群众通过法律途径解决矛盾纠纷，群众“信访不信法”的现象得到明显改善。

（九）政府自身建设持续加强。坚持以法治思维和法治方式推进工作，坚决做到“法定职责必须为、法无授权不可为”。制订了政府机构改革实施方案，加快推进了职能转变和简政放权。石化工业园区与白碱滩区实现一体化管理，工商质监行政管理体制改革划转、三证合一等工作全面完成。简化办事程序，完成市本级行政许可事项确认，继续实施行政许可事项168项、下放16项，实现了行政审批事项减少35%以上的目标。牢固树立过“紧日子”的思想，严控“三公”经费支出，同比降低28%。深入推进“三严三实”专题教育，政府机关作风持续转变。

在总结成绩的同时，我们还应直面发展中存在的问题和困难：一是在新常态下，传统产业优化升级和新兴产业创新发展的步伐还不够快，产业多元化发展的力度还需继续加大。二是作为石油工业城市，克拉玛依既是产能大户，也是耗能大户，节能降耗任务十分艰巨，需要在创新发展、结构优化上不断努力。三是城市精细化管理“大联动”体制机制虽已初步建立，但一些部门重视程度不够，在推进过程中还存在推诿扯皮现象，各区城市管理主体责任还需强化，数字化平台建设还需加快。四是全社会维稳合力尚未完全形成，在工作推进中，一些部门和领导还存在畏难情绪和不敢讲、不会讲的问题。五是政府机关有些部门主动作为、开拓创新意识不够，效率不高。对于这些矛盾和问题，我们将高度重视，在今后的工作中认真加以解决。

今后五年和2016年的工作部署

今后五年，是加快推进世界石油城建设的重要时期。中央关于“一带一路”和中巴经济走廊建设的战略布局，自治区关于打造丝绸之路经济带核心区的决策部署，中石油集团公司关于建设“新疆大庆”的发展举措，为我们开放发展开辟了广阔空间。第二次中央新疆工作座谈会以来，国家、自治区作出的一系列决策部署和政策措施，中石油集团公司支持力度的加大，上海市人才援克的持续深入，为我们创新发展创造了良好环境。我们要强化机遇意识、改革意识、创新意识，切实把思想统一到市委的决策部署上来，把干劲凝聚到建设美好家园的各项具体工作中来，努力谱写克拉玛依发展的新篇章。

今后五年，政府工作的总体思路是：深入贯彻党的十八届五中全会、自治区党委八届十次全委（扩大）会和市油田党委十届七次全委会的重大部署，在市委的坚强领导下，坚定信心，坚持一张蓝图绘到底，围绕社会稳定和长治久安的总目标，以丝绸之路经济带核心区和中巴经济走廊建设为契机，以能源资源开发和信息、金融、旅游产业为抓手，加快建设丝绸之路经济带核心区石油中心，打造区域中心城市，努力在全疆率先基本实现现代化，为新疆社会稳定和长治久安做出更大贡献。

主要奋斗目标是：到2020年，力争实现地区生产总值1200亿元左右，年均增长7%。地方生产总值实现年均增长13%以上，占GDP的比重达30%；服务业增加值年均增长12%，占GDP比重进一步提高，达到35%；全社会固定资产投资规模累计实现2200亿元以上，年均增长12%；社会消费品零售总额实现90亿元，年均增长9%；一般公共预算收入105亿元，年均增长7%；城镇居民人均可支配收入和农牧民人均纯收入与自治区保持同步增长。

为实现上述发展目标，我们必须坚持以下原则：

一是坚持创新发展。创新是引领发展的第一动力。作为典型的资源型城市，我们要把创新摆在发展全局的核心位置，把创新作为推动科学发展的根本动力。结合克拉玛依发展特点，更加关注新能源领域的变化变革，充分发挥资源和人才优势，坚持以科技为引领，深入实施创新驱动，培育发展新动力，拓展发展新空间，构建产业新体系，提升城市可持续发展能力。

二是坚持协调发展。作为组团式城市，结合各区实际和特色，走差异化发展之路，是推进全市整体协调发展的重要举措。各区要根据发展定位，创造性地开展工作，实现优势互补、协同发展。同时，要处理好地方与驻市央企、兵团之间的关系，在科技创新、资源开发、环境治理、节能降耗等方面，探索建立合作新机制，共同建成兵地油融合发展示范区。

三是坚持绿色发展。围绕建设美丽克拉玛依的目标，切实把生态文明的理念融入到经济社会发展的各个方面，结合克拉玛依戈壁荒漠生态的实际，扎实推进生态文明建设，构建科学合理的生态安全格局，促进人与自然和谐共生。加快建立绿色低碳循环发展产业体系，探索发展新能源、新材料等节能环保产业，实现经济效益、社会效益和生态效益的有机统一，增强发展的可持续性。

四是坚持开放发展。充分发挥克拉玛依毗邻中亚、俄罗斯等地区的地缘优势，推动与丝绸之路经济带沿线各国的全方位务实合作，实施“走出去”战略，发展外向型经济，进一步拓展发展空间，着力打造区域中心城市。加强北疆西部区域产业合作、市场融合、设施共建共享，推动区域经济一体化发展，努力成为新疆经济发展新的增长极。

五是坚持共享发展。把持续保障和改善民生贯穿经济社会发展始终，加强民生薄弱领域和薄弱环节建设，构建公平公正、共建共享的新机制。坚持居民收入和经济增长同步，增加更多高质量就业岗位，让发展成果更好地惠及全体市民，实现富民与强市的有机统一，进一步提升市民的获得感和幸福感。

六是坚持和谐发展。没有稳定，就没有发展。要围绕社会稳定和长治久安总目标，全力夯实社会稳定基础，着力推进依法治市进程，为经济社会发展创造和谐稳定的环境。

2016年，是“十三五”开局之年，做好今年各项工作意义特别重大。根据市油田党委十届七次全委会精神，2016年政府工作的总体思路是：围绕打造世界石油城的战略部署和“十三五”规划，以深化改革为动力，以创新发展为引领，坚持石油石化核心产业和新兴产业同步发展、协同推进，继续推进高品质城市建设和精细化管理，大力发展各项社会事业，持续改善民生，全力维护稳定，加速政府职能转变。通过全市共同努力，产业优化升级和多元化发展持续推进，城市品质进一步提升，市民生活水平再上新台阶，产、城、人三者和谐共进、持续向上、充满活力。

主要发展目标是：实现地区生产总值（GDP）增长7%；地方生产总值增长10%；第三产业增加值增长11%；全社会固定资产投资与上年基本持平；一般公共预算收入增长1.3%；社会消费品零售总额增长8%；居民消费价格指数涨幅控制在3%以内；城镇登记失业率控制在3%以内；城镇居民人均可支配收入和农牧民人均纯收入与自治区保持同步增长；努力完成节能减排任务。

根据总体思路和发展目标，重点抓好以下工作：

一、全力推进石油中心建设，促进经济稳定增长

建设六大基地，发展三大新兴产业，促进产业的多元化发展，是我们保持战略定力，增强战略自信，推进资源型城市转型的战略举措，必须坚定不移地向前推进。在经济发展新常态下，更需要通过深化改革、创新驱动和开放发展，促进传统产业优化升级、提质增效，促进新兴产业创新发展、多元发展，实现可持续发展。

（一）力促油气核心产业提质增效。

保持油气生产平稳运行。支持各油田企业加大勘探力度、掌控油气资源、科学有效开发，完成生产经营任务，扩大生产能力。力争全年实现原油产量1281万吨，天然气产量28亿立方米，辖区内原油产量966.5万吨。其中，新疆油田公司生产原油1181万吨，辖区内实现876.5万吨；新春采油厂生产原油100万吨，辖区内实现90万吨。力争年内完成新疆油田公司属地法人注册与合资合作改革。支持黑油山公司落实20万吨原油年度产量、富城能源公司2亿立方米天然气回收任务，帮助企业解决区块审批、边零井管理、销售渠道等问题。落实油砂矿投资，寻求合作伙伴，探索通过抵押贷款、转让等方式，盘活现有存量资源。

力推炼油化工高效发展。积极争取中石油集团公司支持，推动独山子石化公司乙烯原料轻质化项目、克拉玛依石化公司超稠油加工技术改造及油品提升项目；启动两大石化公司成品油国五改造项目；在确保独山子石化公司和克拉玛依石化公司原油加工量不低于780万吨和570万吨的前提下，配合争取更多的加工量。协调中石油西北销售公司解决两大石化公司产品销售问题，畅通产品销售后路。年内完成独山子石化公司属地法人注册与合资合作改革。扎实做好克拉玛依石化园区申报国家高新技术产业开发区工作。

增强技术服务实力。围绕新疆油田工程技术服务企业资质认定管理中心建设，协调中石油、中石化认可地方资质评定标准，为地方企业开拓外部市场提供支持。支持科力、贝肯等企业扩大在哈萨克斯坦、伊朗、蒙古等国开展技术服务业务，提高海外市场份额。发挥新疆石油技术服务企业联合会作用，引导地方企业抱团开拓海外市场，鼓励并支持地方企业由单一工程技术服务企业向综合性油气服务公司发展。

扩大机械制造规模。打通石油装备制造产业链，做大、做实机械制造产业。协助符合条件的地方企业争取中石油一级物资供应商资质，尽快释放产能，带动相关配套企业发展。引导成立机械制造企业协会，组建机械产品质量检测认证中心和石油机械装备技术研究中心，不断提高装备企业和石油企业联合研发、联合开拓市场的能力。

提高油气储备能力。依托中石油、中石化集团公司在疆油气勘探开发建设和低油价的有利契机，争取增加新的石油储备库项目，提高呼图壁储气库储气能力，不断扩大油气储备基地规模。

完善工程教育体系。全力支持中国石油大学（北京）克拉玛依校区运行，加大克拉玛依职业技术学院、克拉玛依技师培训学院招生和培训工作力度，积极开展好宣传工作，不断扩大知名度和影响力。整合工程教育基地教育资源，探索“政产学研”合作模式，助力城市创新发展。积极构建从专科到专业学位研究生工程技术学历教育、从初级工到高级技师培训与资格认证教育的完整教育体系，打造丝绸之路经济带国际化人才培养的重要基地。

（二）力促新兴产业开放创新发展。

大力提升信息产业发展质量。落实“互联网+”行动指导意见，以开放的理念推动互联网与能源、金融等产业结合，实现优化升级。着力研究云计算、大数据的需求特点和发展模式，以市场需求拉动产业健康可持续发展。积极争取中国电信（新疆）数据中心、浪潮数据中心、国家部委数据备份中心、援疆省市灾备中心尽快落户我市。推进国家电子政务外网西北数据中心和国家“一带一路”大数据中心项目建设，实现自治区数据异地灾备中心、中国移动（新疆）数据中心上线运行，形成数据中心规模优势和成本优势。支持政务云、医疗服务向“四地五师”全面推广，形成可复制、可推广的信息系列化产品，提高信息产业效益。全年信息产业增加值力争实现30%的增长速度。

积极推动金融产业创新突破。加快制定完善金融发展政策，推动昆仑银行和中石油专属财产保险公司加快发展，进一步拓展业务范围、扩张发展规模。争取国家和自治区的支持，扩大中亚商品交易中心在线交易业务范围，推进乙烯、沥青、润滑油等大宗化工产品线上交易，不断扩大交易规模。丰富金融业态，积极引进各类金融机构，开展金融合作，增大金融体量。研究组建产业投资基金，持续参与多层次资本市场建设，重点推进贝肯能源、新油股份上市，引导更多地方企业在全国股转系统和新疆股权交易中心挂牌。全年金融产业增加值力争实现16%的增长速度。

着力促进旅游产业开放发展。坚持“景城一体”和“开放合作”的理念，不断完善旅游产业链。围绕国家级旅游业改革创新先行区创建，积极争取国家政策支持，加快旅游机制体制创新，激发产业要素活力，打造智慧旅游，助推旅游产业转型升级。启动石油博览园项目，打造以石油博览、文化体验为主题的旅游综合体。加强与周边地区的旅游合作，推动独山子大峡谷景区开发。以兵地融合发展为契机，整合乌尔禾旅游资源，制定乌尔禾旅游实验区发展规划，推进魔鬼城国家5A级旅游景区创建，加快推进摄影书画小镇、影视文化旅游基地建设，增强旅游核心吸引力。全年旅游收入力争增长15%。

（三）力促现代农业特色高效发展。

积极筹建农业投资开发公司，推动绿成公司混合所有制改革，激活现代农业发展内生动力。以现代畜牧业服务经济为重点，支持肉牛肉羊奶牛标准化、规模化养殖场建设，拓展畜牧业全产业链功能。按照“当地注册、克拉玛依建厂”的思路，稳步推进“四地五师”现代农业融合发展产业园建设。加大“菜篮子”和“果盘子”供给保障力度，打造万亩标准化蔬菜瓜果生产基地。加快市级农产品检测中心建设，完善推广农产品二维码追溯体系，强化畜产品屠宰行业监管职能，从源头上保障农畜产品质量安全。

（四）力促产业多元化发展。

进一步拓展发展领域。紧抓国家、自治区鼓励产业发展的政策，重点关注化工新材料、碳纤维材料、动力电池等科技前沿技术领域，开展有针对性的招商引资工作，提升招商引资质量。积极推进高端化工产业发展，鼓励引导科研机构和企业加大对合成新材料的研究力度，力争实现突破。通过建立孵化基地，探索灵活的产学研合作和产业基金支持体系，发展咨询、产权交易、科研（培训）、中介服务、会展等服务机构。进一步改善消费环境，引导和促进医疗、教育培训、健康养老等服务业发展，构建灵活、公开、透明的政策体系，完善产业发展生态系统，为企业健康发展提供良好的商务环境。

进一步扩大合资合作。推进多种资本参与石油石化领域合资合作。以国有资本为撬动、市场为杠杆，充分发挥社会资本的创造力，吸引战略投资商参与云计算、互联网金融、新材料等领域的项目合作，繁荣地方经济发展。大力推动非公有制经济加快发展，引导非公企业通过出资入股、收购股权、股权置换等方式，参与国有企业改革重组或国有控股企业经营管理。在拓展发展空间、扩大对外合作、推动转型升级等方面，给予非公企业更大支持，充分激发和释放非公经济的活力与创造力，在转方式、促增长、扩就业、惠民生中发挥更大作用。通过政府主导与社会资本合作模式（PPP模式），鼓励和引导社会资本参与基础设施等领域的投资，推进投资主体多元化，促进地方经济发展。

进一步开拓外部市场。加快实施优势产业输出战略，引导地方工程技术服务企业积极参与新疆石油天然气勘查区块招标。推广新疆油建等企业“走出去”参与油田合作的成功经验，依托人才和技术优势，鼓励更多地方企业走出去开拓外部市场，参与购买境外低效油气田。全力支持中亚科技吉尔吉斯斯坦油气合作项目，鼓励更多有实力的地方企业在阿富汗、吉尔吉斯斯坦、哈萨克斯坦等国家开展项目开发。借助新疆克拉玛依论坛的影响力，加快巴基斯坦瓜达尔港克拉玛依园区建设，加大园区招商力度。

进一步服务企业发展。用好用活国家和自治区在降低社会保险费、税费负担、财务成本、电力价格、物流成本等方面的政策措施，千方百计帮助企业降低成本。优化企业发展环境和投资环境，鼓励和支持各种所有制企业创新发展，提高投资信心，改善市场预期。加大企业帮扶力度，积极开展企业负责人培训，培养企业家精神。引进一批市场前景广阔、竞争优势突出的高端产业项目，培育一批创新型高新技术企业，提升企业内在品质。积极鼓励“政银企”合作，着力化解企业融资难题，促进经济持续健康发展。

进一步推进大众创业、万众创新。探索建立创业引导基金，建立健全创业创新综合服务体系，支持建设创业基地、大学生创业园、科技企业孵化器和新型众创空间等创业创新服务平台，鼓励更多资源参与大众创业、万众创新，带动新兴产业、新型业态快速发展。加快建设小微园区，通过政策支持、科技帮扶，协助小微企业向专业化、精细化转变，逐步打造涵盖龙头企业、小微企业，特色鲜明、协作紧密的产业链条。

（五）以全面深化改革增添发展动力。

坚持稳中求进、改革创新、提质增效，加快推进各领域改革逐步深入、见到成效。开展油气管理体制和央企公共服务业务市场化改革，建立完善地企技术转移、合作体系，实施国有企业分类管理。加快政府职能转变，注重简政放权、放管结合和优化服务相统一，降低企业制度性交易成本。扎实推进社会事业领域改革，健全促进大众创业、万众创新的政策体系，加快构建现代学校制度，落实公立医院改革举措，完善公共服务体系。完善城市管理体系，深化行政执法体制改革，继续推进生态文明、文化建设、新型城镇化等领域改革，确保改革任务有效落实。

（六）以创新驱动促进产业转型升级。

大力弘扬克拉玛依改天换地、创新发展的优良传统，以建设创新驱动发展试验区为目标，大力实施创新驱动发展战略，联合大连化物所等国内外科研机构组建创新平台，依托传统优势产业和战略性新兴产业龙头企业，推进化工新材料、能源化工、新能源等新兴产业发展。加强政策对接，引导本地企业加大与央企、沪企的合作力度，实现产业链招商新突破。以科技创新为引领，积极推动“双创”与“互联网+”、“中国制造2025”相结合，形成大众创业、万众创新的新生态系统。继续加大人才引进和培养开发力度，营造开放包容的创新发展环境。鼓励本土人才脱颖而出，加强技能型人才培养。积极支持科技型中小企业做优做强，扩大规模产出效益。加强知识产权运用和保护，激发全社会创新热情和创造活力。

（七）以交流合作推进开放包容发展。

以友好城市和世界能源伙伴组织为载体，进一步拓展国际合作领域，开拓更为广阔的发展空间。发挥好境外联络处的平台作用，引导和帮助企业拓展境外业务，开展多种形式的合作，组团开拓国际市场。举办好第四届信息化创新克拉玛依国际学术论坛暨IT设备技术展，办好新疆克拉玛依论坛、北疆西部区域融合发展研讨会、首届丝绸之路（克拉玛依）国际科学节、2016新疆国际旅游商品博览会等大型展会活动，力争办出品牌，为我市的开放发展搭建交流合作平台。

二、持续推进高品质城市建设，提升城市发展内涵

发挥规划引领和土地保障作用。按照精品规划、精品设计的原则，依据城市总体规划，加强城市外围生态建设，启动编制戈壁生态建设规划，加快编制和完善供水、供热、供电等专项规划和重要区域控制性详细规划，塑造具有城市特性的建筑文化和空间特色，为城市发展提供保障。完善土地收储制度，科学调整科研及产业用地性质，强化土地用途管制，构建节约集约用地战略布局。

深化城市精品建设和精细管理。加快推进厚博学院、工程教育基地、云计算产业园、金沟河引水工程等重点项目建设，启动医学基地建设。实施丰源路、兴生路、玉缘路等道路设施建设。做好国际学校建设、三平水库扩建、地下综合管廊建设等重点项目的前期工作。通过落实精品规划、精品设计的构思和创意，力争将每项工程都打造为精品工程。初步完成城市管理数字平台建设，实现城市精细化管理的实时监督、高效考核。全面推进街道社区精细化管理试点工作，努力探索符合克拉玛依实际的城市精细化管理工作经验。

持续推进生态环境建设。完成金龙湖公园、白碱滩怡心园、独山子城市公园等29项“大绿化”工程建设任务，新增绿地5800亩。继续深入开展“蓝天工程”建设，建立健全污染源档案，对污染物指标控制实行量化管理，全部淘汰2005年底前注册运营的黄标车。大力开展油库、加油站、油罐车油气回收治理，建立环境、气象数据共享机制。2016年，力争全市空气质量优良率达到92%以上。

三、加强社会建设和民生改善，提升市民生活品质

全力提升教育品质。全面深化教育领域综合改革，加快推进“分级管理、以区为主”的管理体系，逐步实现全市中学划归各区管理，进一步理顺区域教育管理体制。开展“教育品质提升年”活动，深化特色学校建设，全面提升教育品质。深入推进“管办评”分离改革，探索实施“第三方评价”的教育评价体制机制，健全教师激励机制，建立中小学校长职级制，加速推进教育治理体系和治理能力现代化。深化双语教育，积极稳妥推进统一编班，促进教育优质同步发展。

加强医疗卫生工作。全面推进公立医院改革，完善医保支付、药品采购和薪酬管理等制度。加快医疗信息化的推广应用，提升医疗健康产业的辐射带动能力。鼓励社会力量参与医学基地和妇女儿童医院建设。积极接入上海优质医疗资源，强化医疗人才引进和培养。加强公共卫生机构建设和运行管理，提高公共卫生服务水平。坚持计划生育基本国策，全面落实二孩生育政策。以国家居住证制度改革为契机，探索促进人口规模与城市发展相适应的适度增长机制。进一步加强流动人口计划生育管理。强化食品药品监督管理，提高食品药品安全水平。

提升就业和社保水平。坚持就业与产业发展相结合，建立就业与产业的良性互动机制，大力推进“双创”工作，制定并实施积极的就业创业政策，引导和推动就业市场多元化发展。积极拓宽就业领域，多渠道开发就业岗位，突出做好高校毕业生就业服务和就业困难人员帮扶安置，全年提供就业岗位5000个以上，实现新增就业再就业2500人以上。完善社会救助体系，建立居民家庭经济状况核对机制，逐步实现救助精准化。鼓励社会力量参与养老产业，着力构建旅游、养老、医疗三位一体的养老服务体系。

繁荣文化体育事业。以现代文化为引领，大力发展文化体育事业，扶持优秀文化产品创作生产，为改革发展稳定凝心聚力。完成国家公共文化服务体系示范区创建。建设区域性文化数字平台，组织百日文化广场、全民健身等群众文化体育活动，推进公共文化服务向纵深发展。创新发展文化体育产业，积极策划引入文化体育精品赛事，举办“五月中国”——中美国际艺术节、国际街舞大赛等文化艺术交流活动，培育本土文化体育市场，推动文化体育和旅游产业融合发展。

优化市民出行环境。争取开通克拉玛依—上海航线，积极推进“空地联运”。完成中心城区公共交通专项规划，适时调整和完善连通家居建材城、汽车城等商业区和绿色康城、城南小区等居住区的公交线路，方便市民出行，促进宜居宜业协调发展。

加强居住环境建设。完善住房保障体系，适时启动限价商品房建设，新建公租房721套、棚户区改造90套，满足中低收入居民住房需求。优化房屋设计，提高房屋品质，加大高品质住房建设力度，挖掘市场潜力。推进小区停车位规范管理，继续引导住宅小区物业服务价格形成机制市场化改革。

提升公共服务能力。在城市管理、工程招投标等领域全面推广诚信体系应用，扩大联合惩戒平台使用范围。完善标准化公厕监督考核标准，切实提高公厕标准化管理水平。加大政府向社会购买服务力度，规范购买程序，健全综合评审机制，提升社会组织承接政府购买服务能力。

依法加强安全监管。牢固树立安全生产“红线”意识，全面落实安全生产责任制和政府安全监管职责，坚决遏制防范重特大事故发生，促进全市安全生产形势持续稳定。进一步优化应急处置流程，加强与驻市央企的应急联动，加速资源整合，提升应急救援处置能力。

四、全力抓好安全稳定，维护和谐社会环境

五、加强政府自身建设，推进治理体系和治理能力现代化

坚持依法行政。自觉运用法治思维和法治方式深化改革、促进发展、维护稳定，推进政府治理体系和治理能力现代化。探索开展政府立法和规范性文件评估，确保制度建设质量。完善重大事项决策的风险评估、合法性审查、集体讨论决策程序，严格依法办事，规范行政权力运行。强化对行政权力的监督制约，坚持在市委领导下，自觉接受人大、政协和社会监督，让权力在阳光下运行。

加快职能转变。理顺部门权责关系，厘清职责交叉和分散事项，强化机构和职责整合。制定市本级部门行政权力清单和责任清单，建立“权界清晰、权责一致、运转高效”的政府职能体系和科学有效的权力监督、制约、协调机制。以大部制改革为目标，优化人员、资源、机构配置，进一步提高政府部门运行效率和工作质量，降低服务对象成本，更好地服务经济社会发展。

突出作风建设。以“三严三实”专题教育为抓手，加强对中央、自治区有关政策的学习，进一步提高政策理解力、执行力和落实力，坚决杜绝不懂政策乱干工作、违反规定乱作决策现象。强化绩效管理、效能监察和行政问责，大力整治行政不作为、慢作为、乱作为现象，打通服务群众“最后一公里”，提高政府执行力和公信力。加强政府窗口部门服务管理，做到单一窗口受理，实现在线审批。树立亲商、安商、富商服务理念，以最真诚的合作态度、最优质的创新创业环境，让企业家们安心创业、乐居生活。

坚持廉洁从政。严格执行中央八项规定、党风廉政建设责任制和党员廉洁自律准则、领导干部廉洁从政准则等规定，筑牢思想防线。深入实施政务公开、信息公开，全面实施“三公”经费公开。认真执行领导干部报告个人事项、重大事项请示等制度，确保干干净净做事、清清白白做人。树立过“苦日子”的思想，加强财政预算和债务管理，严肃财经纪律，做好风险防控，提高资金使用效率。

各位代表，“十三五”宏伟蓝图已经绘就，2016年各项目标已经明确。让我们紧密团结在以习近平同志为总书记的党中央周围，在自治区党委、政府的坚强领导下，按照市委的部署，紧紧抓住历史机遇，勇敢面对各种挑战，团结一致，奋力拼搏，努力实现“十三五”良好开局，为推进世界石油城建设、谱写全市人民幸福美好生活的新篇章而努力奋斗！
